# Population, biomass, and economic value of small ruminants in Ethiopia

**DOI:** 10.3389/fvets.2022.972887

**Published:** 2022-10-13

**Authors:** Wudu T. Jemberu, Yin Li, Wondwosen Asfaw, Dianne Mayberry, Peggy Schrobback, Jonathan Rushton, Theodore J. D. Knight-Jones

**Affiliations:** ^1^Global Burden of Animal Diseases Program, Institute of Infection, Veterinary and Ecological Sciences, University of Liverpool, Liverpool, United Kingdom; ^2^International Livestock Research Institute, Addis Ababa, Ethiopia; ^3^Department of Veterinary Epidemiology and Public Health, University of Gondar, Gondar, Ethiopia; ^4^Commonwealth Scientific and Industrial Research Organization, Agriculture and Food, St Lucia, QLD, Australia; ^5^Independent Consultant, Addis Ababa, Ethiopia; ^6^Institute of Infection, Veterinary and Ecological Sciences, University of Liverpool, Liverpool, United Kingdom

**Keywords:** biomass, economic value, herd model, population, production, small ruminants

## Abstract

Ethiopia has a large population of small ruminants (sheep and goats) which are mostly kept in traditional subsistence production systems that are poorly described. Understanding these different systems, their population structure, biomass, production, and economic value is essential for further analysis and effective policy making. The objective of this study was to quantify these parameters for small ruminant production systems in Ethiopia to use them as a basis for analysis of disease burden within the Global Burden of Animal Diseases program. Population structure and trends of small ruminants were analyzed using data from ten annual national agriculture surveys. A stochastic herd model was used to simulate the small ruminant population, biomass, and economic value. The model was parameterised stochastically using data from statistical databases and the literature, and sensitivity analysis of main model outputs to the stochastic inputs was done. Small ruminants are held across the country mainly managed under two major production systems: the crop-livestock mixed system and the pastoral system. The small ruminant population has grown in the past 10 years with an average annual growth rate of 4.6% for sheep and 6.7% for goats. The national average small ruminant population for 2021 was projected at 96.4 (range 95.3–97.7) million heads and the mean stock biomass was about 2,129 (range 1,680–2,686) million kilograms. The monetary value of the small ruminant population was estimated at USD 5,953 (range 4,369–7,765) million. The annual monetary value of small ruminant production outputs was estimated at USD 1,969 (range 1,245–2,857) million. Although the small ruminant population is large and rapidly growing, contributing about 2% of national annual GDP, the sub-sector is characterized by low productivity, low offtake rates, and a limited range of production outputs with no signs of intensification. Efforts should be made to reduce small ruminant mortality, improve fertility, and better utilize products such as milk to improve the livelihoods of rural households and to benefit the national economy. The approaches developed in this study can be replicated in other systems and countries to reveal trends in the size and value of livestock systems, providing a better understanding of its economic importance and performance.

## Introduction

Ethiopia has one of the largest populations of small ruminants (42.9 million sheep and 52.5 million goats) in the world (1). Based on livestock population statistics available in FAOSTAT, this accounts for around 10% of Africa's and 4% of the world's small ruminant population (2). Yet, these animals and the systems they are kept in are poorly studied.

Small ruminants play an important role in food security for livestock keeping households (3), with small ruminant ownership ranging from 11 to 60% of households in the highland mixed agriculture regions and 41–95 % in lowland pastoral regions of the country (4, 5). The popularity of small ruminants reflects their many benefits: they require low capital investment, and have short breeding cycles and fattening times, and can produce multiple offspring each year. This provides a faster return on investment compared to cattle, can assist with short term cash flows, and helps flocks recover from drought quickly. Small ruminants make up about 25% of the value of meat produced in Ethiopia (6). They also provide significant contributions to the national economy by contributing to export trade. For example, in 2018/19 small ruminants made up about 86% of the USD 93 million meat export revenue and about 8% of USD 45 million live animal export revenue of the country (7).

Despite a large population, small ruminant production in Ethiopia is largely based on traditional subsistence production with limited commercialization and modernization. Both production offtake rates and production quantities are very low (6). Reproduction rates are also low with long age to maturity, long lambing/kidding intervals, and very high young stock mortality (6, 8).

Technical analysis of problems in existing production systems and potential interventions such as feed, genetics and health are needed to bring sustainable improvements in the sector. This requires an understanding of the systems, including its population structure, biomass, production, and economic value. However, this understanding is limited in the small ruminant livestock sector in Ethiopia.

The Global Burden of Animal Diseases (GBADs)^1^, (9) is a research programme which aims to estimate the burden of animal diseases by livestock sectors and production systems to support rational decisions with regard to animal health and production interventions. This paper presents estimates on the scale, distribution, economic value, and trends of the small ruminant sector in Ethiopia to be used as foundation for animal disease burden estimation. The results include important findings relevant to livestock policy in the sector.

## Materials and methods

### Production system classification and mapping

Small ruminant production was classified and mapped into different systems and subsystems to create uniform analytical units. First, sheep and goats were allocated to pre-existing agro-ecological systems based on Shapiro et al. (6) and Sere and Steinfeld (10). These systems were (1) sedentary mixed crop-livestock systems in rainfed temperate and tropical highlands (CLM), (2) nomadic pastoral and agropastoral arid and semi-arid grazing lands (pastoral), and (3) intensive and landless specialized ruminant production systems. The borders of these agroecological based production systems were aligned to administrative zones. Where more than one system was present, the predominant production system was assigned to the whole zone. The regional and zonal administrative map of Ethiopia is provided in Supplementary Figure S1.

The first two systems (CLM and pastoral) were further classified into subclasses according to feeding system practiced and household livestock composition. In this subclassification, the CLM system was divided into two sub-systems based on feeding system (11); small ruminants kept mainly at communal grazing within cereal crop areas, and small ruminants tethered in yards with feed brought to them in the *enset*^2^ growing areas of the country. In this classification, a zone in the CLM system area was classified as “tethered in *enset* growing area” when the intensity of *enset* production in the zone had an *enset* tree to small ruminant ratio of ≥ 1 (1, 12). In *enset* growing areas in southern Ethiopia, small ruminants are mainly kept under tethering/semi intensive management system (11). The pastoral system was also divided into two sub-systems based on household livestock composition; those where the dominant species owned by a household was small ruminants and those where the dominant species in the herd was cattle, based on tropical livestock units (TLU) contribution in the herd.

The production systems mapping was done using QGIS 3.18 GIS software (QGIS Geographic Information System. Open-Source Geospatial Foundation Project. http://qgis.osgeo.org).

### Analysis of small ruminant population patterns

The temporal trend of the small ruminant population (2011–2020) was analyzed using data from the CSA annual agricultural sample surveys^3^. Annual and ten-year population growth rates were calculated by production system and species, with population growth analyzed in terms of growth in flock sizes and number of flocks. The national annual agricultural sample surveys covered only two out of five administrative zones from Afar region until 2018, and only three out of eleven administrative zones from Somali region until 2019. The small ruminant population in these regions for the previous years were, therefore, adjusted to account for this based on the proportion of small ruminants in the uncovered zones derived from the 2019 and 2020 surveys (population proportions in the zones were roughly stable through time) using the following formula.


$$\begin{array}{l} P_{adj}=P+\left(P*Pr_{excZP}\right) \end{array}$$

Where, P_adj_ is the adjusted population for a previous year, P is the population in a previous year and Pr_excZP_ is the average proportion of the population of previously excluded zones in the latest two years where all zones were included.

Spatial distributions of the small ruminant population were mapped using zone-level number of small ruminants per capita (as the lower scale livestock data available are zone level), calculated as small ruminant population divided by human population, with human population data obtained from CSA (13). Flock structure (size and composition) was analyzed by species and production systems using the latest CSA data set (14).

### Estimating biomass, production, and economic value

#### Herd model simulation

Biomass, production, and economic value of sheep and goats in the different production systems were simulated using the DYNMOD herd model (15). DYNMOD is a spreadsheet based simple herd growth model for ruminant livestock populations, which simulates the dynamics of the population size, and the number of animals produced per year using simplified demographic equations. DYNMOD can also be used to calculate livestock biomass; production of milk, wool, manure, and skins at population level; the financial equivalent of the biomass and production outputs; and crude estimates of feed requirements in dry matter (Supplementary Figure S2).

This herd model was used to simulate and estimate the small ruminant population, liveweight biomass in kilograms (kgs), and production outputs and their financial values for the year 2021. These are calculated by multiplying the average parameter values (live weight, production outputs, prices) in a particular sex-age category by their corresponding numbers in those sex-age categories. While the model directly calculates monetary value of the stock biomass and primary production outputs such as stock variation and live animal offtakes, in our study the monetary value of secondary outputs such as milk, manure and skins were calculated manually by multiplying the physical quantities of these outputs from the model by their corresponding prices.

#### Model input parameters, stochastic simulation, and sensitivity analysis

The herd model uses several input parameters such as initial population size and sex-age structure, demography parameters (reproduction and death rates), production parameters (live animal offtake rates, liveweights, milk offtake, skin offtake and manure production), and output prices (live animal price, milk price, skin price, and manure price), etc. Most parameter inputs such as parturition rate, twinning rate, death rate, liveweight, and animal prices were inputted in the form of probability distributions to capture uncertainties in the input data. Consequently, the model outputs also take the form of probability distributions reflecting their variability and uncertainty. This allowed a sensitivity analysis to assess how individual input parameters influenced the output. The model was stochastically simulated for 100,000 iterations by Latin Hypercube sampling using @ risk software version 8.2 (Palisade Corporation, Ithaca, NY, USA). A sensitivity analysis was conducted for major outputs such as population size, total stock biomass, production outputs and financial value of the total stock of small ruminants. The change in output statistics method was used for the sensitivity analysis.

#### Input parameters

Small ruminant populations were categorized into six sex-age categories for the model. The age groups were made to align roughly with important life stages; juveniles (unweaned animals, < 6 months old), subadults (weaned animals of marketing age, 6– <12 months old), and adults of reproductive age (1–5 years old for males, and 1–10 years old for females).

Reproductive performance is defined by parturition and prolificacy rates. Parturition rate is the number of parturitions per year divided by the number of females of reproductive age present in that year in the population. Prolificacy rate (average litter size) is the average number of offspring born alive per parturition. Parturition rate was derived from the proportion of births to female adults reported in CSA (1) divided by the average litter size. The proportion of births was modeled using Beta distribution. Prolificacy rates were compiled from values reported by Gizaw et al. (16) and Solomon et al. (17), which were used to form a triangular distribution defined by the minimum reported rate, the median reported rate (as the most likely), and the maximum reported rate (Table 1).

**Table 1:** Input parameters of the herd model.

**Parameters**	**Distribution**	**Distribution parameter values**				**Source**
		**Sheep CLM**	**Sheep pastoral**	**Goat CLM**	**Goat pastoral**	
**Reproduction**						
Ratio of new-born to adult female	Beta (α_1;_ α_2_)^a^	10,115, 500 +1; 2,999, 147 +1	6,57, 227 + 1; 2,716, 142 + 1	9,233, 857 +1; 1,631, 141 + 1	11,861, 108 + 1; 3,880, 682 + 1	(1)
Prolificacy rate (litter size)	Triangular (Mn; ML; Mx)	1.11; 1.27; 1.42	1.11; 1.27; 1.42	1.07; 1.52; 2.07	1.07; 1.52; 2.07	(16, 17)
Parturition rate (%)^b^	Point estimate	59	54	57	50	
**Mortality**						
Female juvenile	Beta (α_1_; α_2_)^a^	85 +1; 857 + 1	107 + 1; 198 + 1	118 + 1; 433 + 1	407 + 1; 644 + 1	(8)
Female subadult and adult^c^	Point estimate	4	4	12	8	
Male juvenile	Beta (α_1_; α_2_)	185 +1; 857 + 1	107 + 1; 198 + 1	118 + 1; 433 + 1	407 + 1; 644 + 1	(8)
Male subadult and adult^c^	Point estimate	12	5	19	18	
**Offtakes (%)**						
Female juvenile	Point estimate	0	0	0	0	(1)
Female subadult	Point estimate	4	2	9	4	(1)
Female adult	Point estimate	4	2	9	4	(1)
Male juvenile	Point estimate	0	0	0	0	(1)
Male subadult	Point estimate	54	12	71	63	(1)
Male adult	Point estimate	54	12	71	63	(1)
**Live wight (Kg)**						
Juvenile	Triangular (Mn; ML; Mx)^d^	11; 12; 13	7; 8.5; 10	11; 12; 13	7; 8.5; 10	(16–18)
Subadult	Triangular (Mn; ML; Mx)	13; 16; 19	13; 16; 19	13; 16; 19	13; 16; 19	(16, 17)
Adult	Triangular (Mn; ML; Mx)	20; 28; 35	20; 28; 35	20; 28; 35	20; 28; 35	(6, 17)
**Prices (ETB** ^**e**^ **)**						
Female juvenile	Triangular (Mn; ML; Mx)	1,100; 1,200; 1,300	650; 750; 850	800; 825; 850	800; 825; 850	http://www.lmiset.gov.et
Female subadult	Triangular (Mn; ML; Mx)	1,800; 1,925; 2,750	1,450; 1,617; 1,783	1,550; 1,950; 2,800	1,375; 1,838; 2,300	http://www.lmiset.gov.et
Female adult	Triangular (Mn; ML; Mx)	1,440; 2,825; 4,500	1,400; 1,530; 2,467	2,140; 3,210; 4,093	1,925; 3,378; 3,800	http://www.lmiset.gov.et
Male juvenile	Triangular (Mn; ML; Mx)	1,067; 1,250; 3,500	750; 1,063; 1,375	900; 1,550; 2,200	1,800; 1,450; 1,170	http://www.lmiset.gov.et
Male subadult	Triangular (Mn; ML; Mx)	2,000; 2,600; 3,140	1,425; 1,667; 2,050	1,800; 3,046; 4,350	1,650; 2,495; 3,000	http://www.lmiset.gov.et
Male adult	Triangular (Mn; ML; Mx)	2,500; 6,000; 8,000	1,800; 3,042; 3,617	3,850; 5,900; 9,000	3,440; 4,600; 5,833	http://www.lmiset.gov.et
**Milk production**						
Milk yield (l/day)	Point estimate	–	–	0.53	0.49	(14)
Lactation length (days)	Point estimate	–	–	129	117	(14)
Proportion adult female used for milk (%)	Point estimate	–	–	6	19	(14)

^a^Beta distribution parameters: α1, success plus one; α2, failure plus one.

^b^Ratio new-born to female adults divided by prolificacy rate.

^c^For the subadults and adults, crude (all age) mortality data of the CSA (1) was used but adjusted by proportionally deducting the juvenile mortality as: adult mortality, [crude mortality–(young mortality rate x proportion of young)]/proportion of adults.

^d^Triangular distribution parameters: Mn, minimum; ML, most likely; Mx, maximum.

^e^ETB = Ethiopian birr which is equal to USD 0.0229 on average in 2021.

The death rate corresponds to the proportion that die over the duration of the entire age class if age class length was ≤ 12 months (e.g., for the juvenile class whose age class length is 6 months, the death rate is per 6 months), or proportion that die over a 1-year period where the age class length was >12 months (15). Death probabilities were mapped as beta distributions (Table 1).

Offtake refers to the net animal offtake, i.e., the animals leaving the flock minus animals entering the flock divided by the total number of animals in the flock. The animals leaving the flock consist of animals sold, slaughtered, or given as gifts, and animals entering the flock consist of purchases and gifts received. The offtake rates correspond to offtake over the duration of the entire age class if the age class length is <12 months (e.g., for subadults whose age class length is 6 months, the offtake is per 6 months) or offtake over a 1-year period if the age class length is equal or >12 months (15). It was assumed that offtakes are limited to subadults and adults. The offtake rates are presented in Table 1.

Liveweight for different species and sex-age categories were compiled from the literature (Table 1). A triangular distribution was derived for liveweight with minimum reported weight as the minimum, the median of the reported weight as most likely, and the maximum reported weight as the maximum value of the distribution. There was insufficient data to separate weights in all sex-age category in the different species and production systems, so in some cases the same values were used for both species and production systems. The same carcase yield or dressing percentage (carcass weight/liveweight) value was also used for all species and sex-age categories in all production systems due to the lack specific information for each category. Literature sources reported dressing percentages that range from 40–45% (17, 19, 20). The median value 42.5% was used as most likely value for triangular distribution for dressing percentage.

The price of a live animal was taken from livestock market information system database of the Ministry of Trade and Industry of Ethiopia from for year 2021^4^. The price data for the different markets surveyed were used to parameterise a triangular distribution defined by mean, minimum and maximum prices for each sex-age category (Table 1).

The milk production parameters required by the model were lactation yield per day and lactation length in days. Milk production was considered only from goats as sheep are rarely used for milk production and were not covered in the CSA surveys. Even for goats only 14% of female adult goats were used for milk production (1). From all adult goats used for milk production 83% of them are found in the pastoral system (14). These proportions are considered when entering milk production parameters. For example, as only 19% of goats in the pastoral system were producing milk and the daily milk yield for a goat being milked is 0.49 liter (14) the daily per animal milk yield for all goats in the pastoral goat model was entered as 0.19^*^0.49 liter. Parameters related to milk production are given in Table 1.

Weight of skin for different age and sex categories was also a model input. The skin of sheep and goats in Ethiopia are often sold per piece not per kg. The piece of skin is changed into kg which was estimated as 1 kg.

The limited literature on manure production from small ruminants indicate they produce dry manure weight of about 1–2% of live body weight daily. The conservative 1%, derived from an Ethiopian study (21) was used to derive the amount of manure (kg) produced from different age categories.

## Results

### Production systems and population distributions

#### Characterizing and mapping production systems

**Figure 1:**
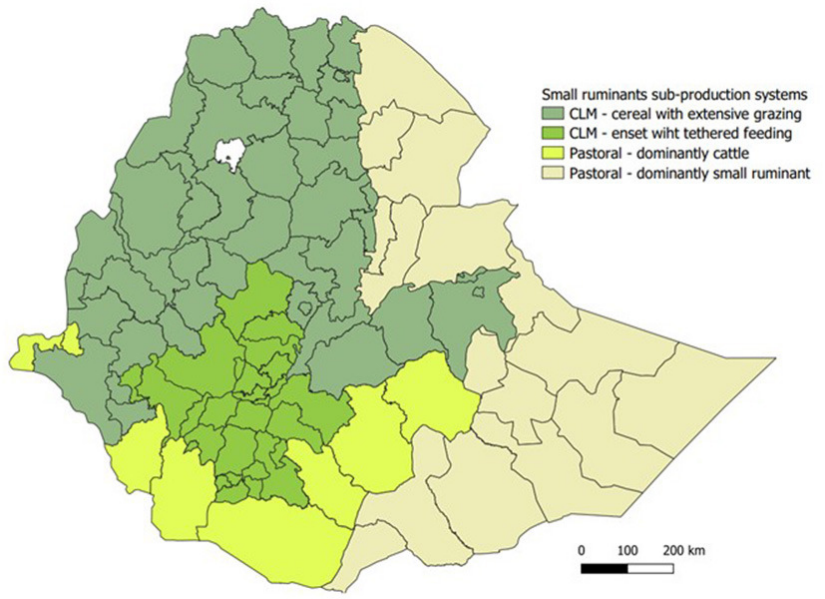
Map of small ruminant production systems. The divisions in the map indicate zones.

The major production systems and sub systems of small ruminant production in Ethiopia are mapped in Figure 1. The detail descriptions of the production systems are provided in Supplementary material S1.

#### Small ruminant populations by species and production systems

The total small ruminant population in 2020 was almost equally distributed between the CLM and pastoral system (Table 2). Species-wise, the CLM system comprised the majority (58%) of the sheep population and the pastoral system in turn contained the majority (58%) of the goats population. From the four subsystems, the pastoral small ruminant dominant system contains most of the small ruminants (43%), followed by the CLM cereal grazing system (38%). Despite the distinction of these four sub-production systems, analyses in subsequent sections of this study are presented at the level of the two major production systems i.e., CLM and pastoral systems because of lack of sufficiently detailed data at the lower levels of classification.

**Table 2:** Distribution of the small ruminant population by production system and species in 2020.

**Production systems**	**Sheep (million head)**	**Goats (million head)**	**Total (million head)**	**Share of national population**
CLM	24.7	22.2	46.8	49%
CLM-cereal growing and grazing	17.8	18.0	35.8	38%
CLM-*enset* growing and tethering	6.8	4.1	11.0	12%
Pastoral	18.2	30.3	48.6	51%
Pastoral- cattle dominant	2.7	5.0	7.8	8%
Pastoral- small ruminant dominant	15.5	25.3	40.8	43%
Total	42.9	52.5	95.4	100%

### The small ruminant population trend, flock size and structure

#### Temporal trend

**Figure 2:**
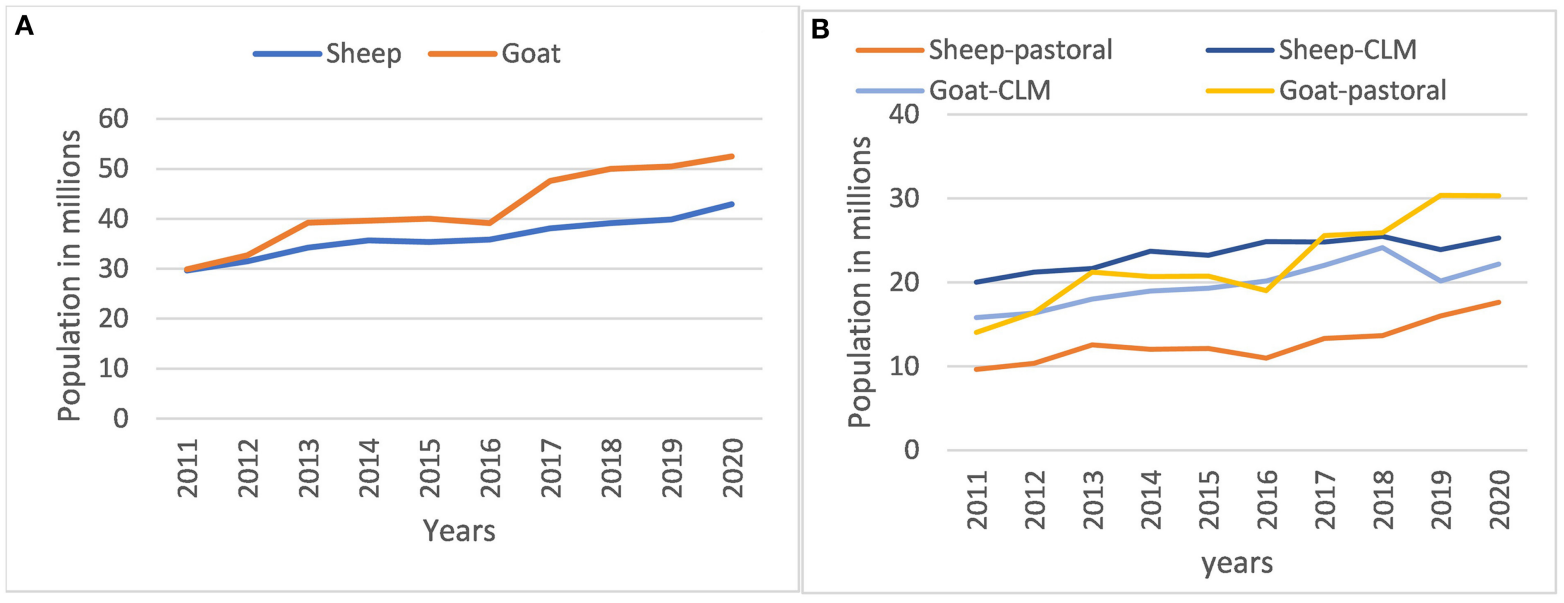
Small ruminant population trends in Ethiopia from 2011-2020, **(A)** by species and **(B)** by species and production systems.

The small ruminant population in Ethiopia grown steadily over the past 10 years from 2011 to 2020 (Figure 2). In this period the sheep population increased by 45% and the goat population increased by 76%. The average annual population growth in this period was 4.2% (range −1.0–8.4%) for sheep and 6.7% (range −0.2–19.2%) for goats. In the first seven years of this period (2011–2017) when the survey coverage was similar and comparable, the increase in population was mainly correlated with an increase in the number of flocks (increase by 20% in sheep and 26% in goats) rather than flock size (increase by 6% in sheep and 12% in goats). The sheep and goats population were about the same at the beginning of the decade, but this has since diverged in favor of goats (Figure 2A). The fastest growth rate happened in goats in the pastoral system (Figure 2B).

#### Spatial distribution

**Figure 3:**
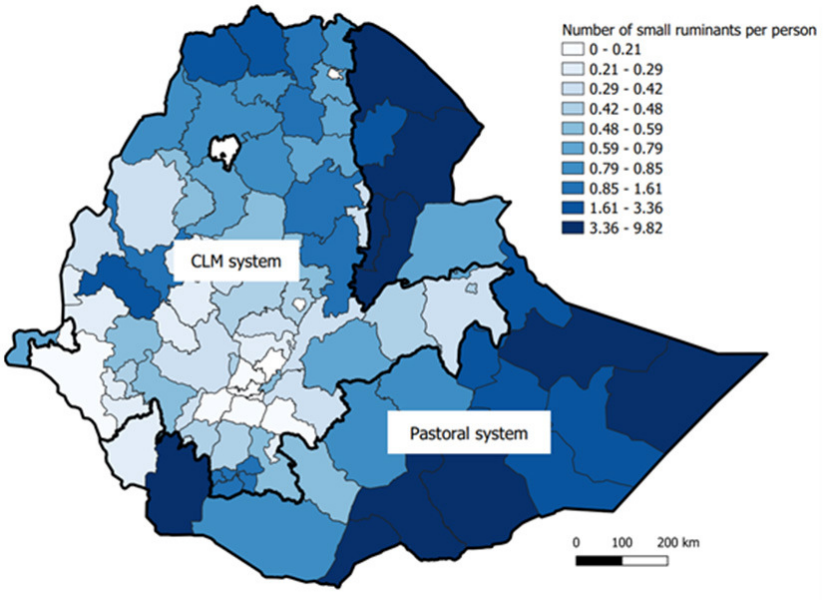
Per capita distribution of small ruminants.

Generally, higher per capita sheep and goat populations were observed in the pastoral regions of the country (Figure 3). However, high sheep number per capita were also observed in some areas of the CLM system such as in the highlands of Amhara and Tigray regions (Supplementary Figure S3A) with high goat number per capita in western lowlands of Tigray region (Supplementary Figure S3B).

#### Flock size and structure

Generally small ruminant flock sizes were small, and their distribution are positively skewed, particularly in the CLM system (Supplementary Figure S4). In the CLM system, the median flock size for sheep was three animals (range 2–83) and for goats four animals (range 1–712). In the pastoral system the median flock size was much larger: 15 animals (range 1–600) for sheep and 20 animals (range 1–714) for goats. In both sheep and goat flocks in the two major production systems, adult females make up more than 60% of the animals (Supplementary Figure S5). This reflects higher offtake rates for males, which are sold for slaughter, while females are generally kept for breeding and milking.

### Biomass and monetary value of small ruminant production

The herd model simulation estimated the average small ruminant population for the year 2021 to be about 96.4 (range 95.3–97.7) million heads and the mean stock biomass as 2,129 (range 1,680–2,686) million kgs which is equivalent to 8.52 million TLU (where 1 TLU = 250 kg liveweight). The monetary value of the total small ruminant stock was estimated at 5,953 (range 4,369–7,765) million USD. The total population, stock biomass, and monetary value of small ruminants by species and production system are summarized in Table 3.

**Table 3:** The simulated number, biomass, and the monetary value of the small ruminant population in 2021.

**Species**	**Production system**	**Population**		**Total biomass**		**Monetary value**	
		**(million heads)**		**(million kg)**		**(million USD)**	
		**Mean**	**^a^Range**	**Mean**	**Range**	**Mean**	**Range**
Sheep	CLM	26.1	26.0–26.1	595	466–722	1,834	1,378–2,417
	Pastoral	18.9	18.5–19.1	454	352–552	1,020	828–1,128
	**Total sheep**	44.9	44.5–45.3	1,049	817–1,274	2,854	2,210–3,645
Goat	CLM	22.3	22.3–22.3	409	363–591	1,413	943–1,938
	Pastoral	29.2	28.5– 30.1	671	500–821	1,687	1,221–2,183
	**Total goat**	51.5	50.8–52.4	1,080	863–1,412	3,100	2,163–4,121
	**Overall**	96.4	95.3–97.7	2,129	1,680–2,686	5953	4,369–7,765

^a^Range: from minimum to maximum of the simulated distribution.

### Production offtakes and economic contribution

Net offtake rates were generally higher in goats (27%) than sheep (18%) and higher in the CLM system than the pastoral system. The monetary value of small ruminant primary production output in terms of increase in stock value and net animal offtake for the whole country in the year 2021 was estimated at USD 1,735 (range 1,065–2,557) million. This value by species and production system is presented in Table 4. The monetary value of secondary production outputs in 2021 such as milk, manure, and skin was estimated at USD 234 (range 180–300) million (Table 5). Overall, small ruminant production in the country contributes about USD 1,969 (range 1,245–2,857) million to the national economy.

**Table 4:** Annual value of primary production (stock variation and net offtake) for year 2021.

**Species**	**Production system**	**Net offtake**		^**a**^ **Stock variation and net offtake**		**Stock variation and net**	
		**rate (%)**		**liveweight equivalent in**		**offtake monetary value in**	
				**millions (kg)**		**millions (USD)**	
		**Mean**	**^b^Range**	**Mean**	**Range**	**Mean**	**Range**
Sheep	CLM	21.9	21.8–22.0	192.7	151.0–231.3	710	451–1042
	Pastoral	13.5	13.3–13.7	96.0	67.7–125.3	228	173–290
	**Sheep total**	18.3	18.2–18.5	288.7	218.7–356.6	938	624–1332
Goat	CLM	26.7	26.0–27.3	129.9	100.8–159.3	446	268–627
	Pastoral	26.6	24.8–28.6	124.2	67.7–210.0	351	173–598
	**Goat total**	26.6	25.3–28.0	254.1	168.5–369.2	797	441–1,225
	**Overall**	22.8	22.0–23.6	524.8	387.2–725.8	1,735	1,665–2,557

^a^Stock variation, The number of animals at the beginning of the year minus the number of animals at the end of a year in the population. ^b^Range: from minimum to maximum of the simulated distribution.

**Table 5:** The annual monetary value of secondary production outputs from small ruminants for year 2021.

**Species**	**Production**	^**a**^ **Milk offtake in**		**Skin in millions**		**Manure in**		**Financial value in**	
	**system**	**millions (liter)**		**(kg)**		**millions (kg)**		**millions (USD)**	
		**Mean**	**^b^Range**	**Mean**	**Range**	**Mean**	**Range**	**Mean**	**Range**
Sheep	CLM	^c^NA	NA	3.7	3.7–3.8	2,173	1,698–2,623	31	25–37
	Pastoral	NA	NA	2.6	2.5–2.6	1,656	1,275–2020	22	17–27
	**Sheep total**	NA	NA	6.3	6.2–6.3	3,829	2,973–4,643	53	42–63
Goat	CLM	30.2	22.1–43.1	6.0	5.8–6.1	438	241–674	42	31–58
	Pastoral	105.7	81.0–137.3	7.7	7.1–8.7	2,400	1,797–3,010	140	107–179
	**Goat total**	135.9	103.1–180.4	13.7	12.9–14.7	2,838	2,038–3,684	181	138–237
	**Overall**	135.9	103.1–180.4	20.0	19.1–21	6,667	5,011–8,327	234	180–300

^a^Milk offtake, milk collected and used for human consumption (i.e., excluding milk consumed by young); ^b^Range, from minimum to maximum of the simulated distribution. ^c^NA = Not applicable.

### Sensitivity analysis

**Figure 4:**
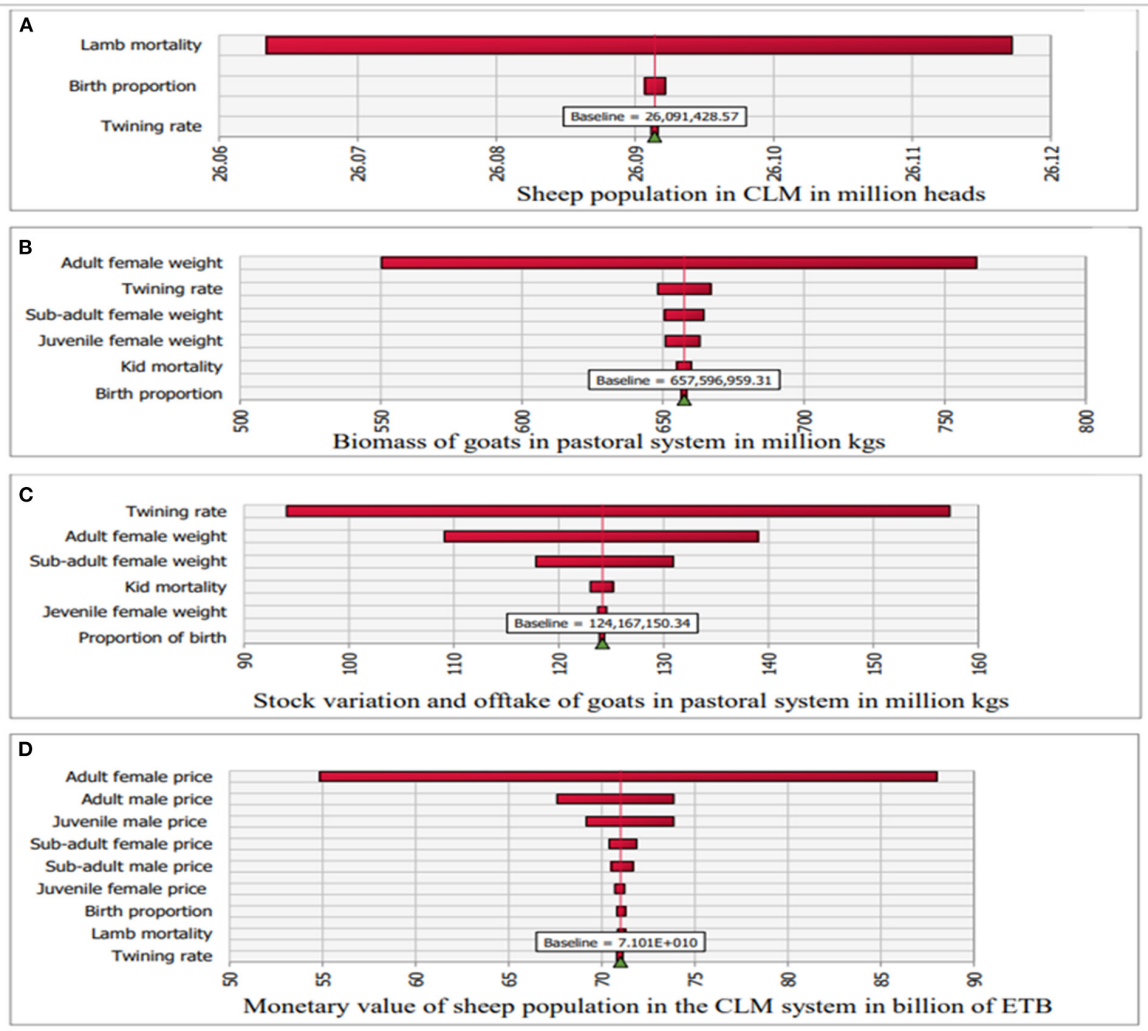
Tornado plots showing sensitivity of model outputs to stochastic inputs: **(A)** Sheep population in CLM in million heads, **(B)** Biomass of goats in pastoral system in million kgs, **(C)** Stock variation and net offtake of goats in pastoral system in million kgs, and **(D)** Total monetary value of sheep population in CLM system in billions of ETB.

In all production systems and both species, except goats in the pastoral system, the population size is strongly influenced by (most sensitive to) lamb/kid mortality (e.g., see Figure 4A for sheep in the CLM system). For goats in the pastoral system, population size is most sensitive to the twinning rate. The estimate of total stock biomass is most sensitive to the weight of adult females in all species and production systems (e.g., see Figure 4B for goats in the pastoral system). The production biomass for small ruminants (stock variation and offtake) was most sensitive to adult female weight in all production systems except for goats in the pastoral system where it was more sensitive to the twinning rate (Figure 4C). The monetary value of the small ruminant biomass was sensitive the live animal prices, being most sensitive to the price of adult females in all species and production systems (e.g., see Figure 4D for sheep in the CLM system).

## Discussion

Ethiopia's sizeable small ruminant population is expanding rapidly. However, there is massive under-performance in part owing to high levels of diseases and hence mortalities. Approaches to quantify key population characteristics are poorly established. Using a stochastic bioeconomic model we simulated the two main small ruminant production systems in Ethiopia. Through this we have shown the key roles that fertility and mortality play in determining the size and productivity of small ruminant systems. This work will now be expanded to quantify and attribute the burden of animal disease on these systems.

Over the past ten-years (2011–2020) the small ruminant population has grown substantially, driven by increases in the number of holdings/flocks rather than flock sizes. This indicates the slow or absence of structural change in the sector toward intensification and commercialization, which should have been manifested by incremental growth in individual flock size and a decrease in the number of holdings.

Similarly, Bachewe et al. (22) found an increase in livestock products that came from an increase in the number of animals rather than increased productivity in the period from 2004 to 2015. This should be given attention in livestock policy if it is to guide the sector toward more specialized and intensive systems with improved production and productivity and reduced environmental impact from extensive over-grazing with increased numbers of animals in traditional systems. The government's 10-year agricultural perspective plan (23), which defined the strategy for small ruminant development through breed, feed, animal health, market improvement, should give emphasis in changing this structural problem in the sector.

While the average small ruminant flock size was small overall, it was larger in the pastoral system than in the CLM system. In line with this, the small ruminant count per capita was higher in pastoral areas. However, high sheep number per capita and high goat number per capita were also observed in the highland zones of the Amhara region and western parts of Tigray region, respectively, in the CLM system. The high count per capita could be an indication of the importance of small ruminants in those areas, which can be prioritized for small ruminant development interventions. It may also reflect the ecological burden from overgrazing of livestock in these areas.

The estimated small ruminant biomass accounts for about 13% of the total livestock biomass of the country and is only second to cattle. This estimate provides insight into the importance of the sector in terms of the resource base and economic contribution. Moreover, the biomass can also be used as a denominator for various livestock related analyses and between species comparisons, such as feed requirements, greenhouse gas emissions and other environmental impacts such as deforestation and land degradation, amongst others.

The herd model predicted net offtakes were higher in the CLM system than pastoral system and for goats than sheep. The offtakes would have been expected to be higher in the pastoral system as livestock production is the main livelihood in this system, but that was not the case. This could partly be due to higher mortality which reaches up to a staggering 45% in lambs/kids and 22% in adults in the pastoral as compared to the CLM system (5, 8). The net offtake rates were generally low (15–30%) but higher than similar net offtakes reported for small ruminants in Ethiopia in the 2000s (24), which were below 10%. In the 2010s a higher gross offtake rate, which reached 40% (25), and lower net commercial offtake rate (below 10%) were reported. However, the interpretation differs for different offtake parameters. Net commercial offtake rate is based only on sale and as such focuses only on commercial offtake, while gross offtake rate does not exclude intakes like purchases and gifts and as such does not indicate the real production output. In our estimates and those of other reports mentioned, the offtake was always higher for goats than sheep which may be the reason the goat production is expanding more than sheep production in the country.

The estimated total economic output from small ruminants is a significant contribution to the national economy which accounts for 2% of USD110 billion GDP estimated for Ethiopia in the year 2021^5^. Much of this was from increased stock value and live animal offtake. The economic contribution of secondary products such as milk and wool was very small compared to its potential, with massive room for improvement. Apart from low productivity, only about a one fifth of goat population was used for milk production. Sheep milk and wool productions were insignificant and were excluded in this estimate. Generally, production from small ruminants can be increased by expanding range of production outputs and improving productivity.

The data used in this analysis have some limitations. The main issue was that available data were mostly crude and not disaggregated by species, sex, or age. When reported by age category, age categories were not uniform. Lamb/kid mortalities were reported for different age lengths. This problem has been also noted in the wider livestock mortality literature globally (26). Furthermore, some parameters were reported by only few studies which may not be representative of the national situation.

Stochastic simulation and sensitivity analysis were undertaken to reflect and investigate the uncertainty in the data. The demographic outputs such as population number were mainly influenced by juvenile mortality. Adult mortality was derived from juvenile mortality, and better mortality data for different age groups would improve the reliability of the demographic outputs. The other main outputs such as stock biomass, production and monetary values were most sensitive to the weight of adult animals, especially female adults. Female adults were particularly important because they constitute the major proportion of the flocks.

Estimates for live weights in the literature varied greatly and this was reflected in the model inputs. One reason for this wide range could be the variability of weight for different local breeds. For example, the 6-month weight of Menz sheep breed is about two-thirds to that of Bonga sheep breed (27). While there are several local breeds of sheep and goats in the country there is insufficient data to allow breed specific analysis.

Various data sources were used, such as the government Central Statistical Agency's (CSA) annual agricultural sample survey and both peer reviewed and gray literature. However, the analysis was heavily based on the CSA data. The CSA data collection is excellent in that it covers the entire country geographically, conducted regularly, and has a good level of granularity in terms of disaggregation by sex, age, and purpose. However, the age disaggregation is limited only to the total population number and is not provided for other important demographic parameters like birth, mortality, and offtake rates. A major weakness of the CSA data is that it is limited to rural holdings. While the commercial sector is a growing sector, no data has been collected or reported for this sector. Missing or inconsistent data was occasionally a problem. In the future, addressing these weaknesses in CSA data will further increase its value for informing national policy in livestock development.

## Conclusions

Ethiopia has a large and rapidly growing small ruminant populations, especially goats. However, the sectors are failing to specialize and intensify. This is needed if output and efficiency are to improve. Despite the high number of small ruminants per capita in the pastoral system, the offtake rates are lower with very high kid mortality up to 40%). Efforts to identify and address this high mortality are needed as this will result in big increases in offtakes and productivity, and thus livelihoods. Small ruminants are underutilized for milk production. Harnessing the large goat population for milk production has the potential to improve nutrition and food security in poor small ruminant keeping households, which is needed given the high levels of undernutrition in rural Ethiopia. Small ruminants constitute about 13% of the livestock biomass in the country and, despite the low productivity, still contribute a sizeable proportion (2%) of annual national GDP. With sound policies and effective implementation this contribution can be greatly enhanced whilst minimizing environmental impact. These findings, and those of follow on analyses on disease burden, are needed to guide future strategies for sustainable growth of the sector.

## Data availability statement

The original contributions presented in the study are included in the article/Supplementary material, further inquiries can be directed to the corresponding author/s.

## Ethics statement

Ethical review and approval was not required for the animal study because the study was based on secondary data.

## Author contributions

WJ designed the study, collected and analyzed data, and wrote the draft manuscript. YL analyzed data. WA collect data. DM and PS wrote part of the manuscript. JR conceived the idea and designed the study. TK-J designed the study, led the study, and wrote the manuscript. All authors contributed to manuscript revision, read, and approved the submitted version.

## Funding

This study was supported by Global Burden of Animals Diseases Program funded by the Bill and Melinda Gates Foundation, Seattle, WA, and Foreign, Commonwealth and Development Office of UK government [Grant number NV 005366) and International Livestock Research Institute funded by CRP livestock of Consultative Group on International Agricultural Research.

## Conflict of interest

The authors declare that the research was conducted in the absence of any commercial or financial relationships that could be construed as a potential conflict of interest.

## Publisher's note

All claims expressed in this article are solely those of the authors and do not necessarily represent those of their affiliated organizations, or those of the publisher, the editors and the reviewers. Any product that may be evaluated in this article, or claim that may be made by its manufacturer, is not guaranteed or endorsed by the publisher.

## Footnotes

^1^ https://animalhealthmetrics.org/

^2^Enset (*Ensete ventricosum*), is a perennial herbaceous tree belonging to the family Musaceae, along with banana and plantain used as a staple food in southern Ethiopia.

^3^Ethiopia Central Statistical Agency: https://www.statsethiopia.gov.et/our-survey-reports/, accessed July 15, 2021.

^4^Livestock marketing information system of Ministry of Trade and Industry of Ethiopia: http://www.lmiset.gov.et/, accessed August 20, 2022.

^5^Trading economics: https://tradingeconomics.com/ethiopia/gdp, accessed December 12, 2021.
