# Supplementary material for: Population, biomass, and economic value of small ruminants in Ethiopia
Source: Front Vet Sci. 2022 Oct 13;9:972887. doi: 10.3389/fvets.2022.972887 (PMC9608676; doi:10.3389/fvets.2022.972887)
Supplement: Supplementary file 1 [file Data_Sheet_1.docx]

**Supplementary Materials**

**S1. Small ruminant production systems in Ethiopia.**

1) Sedentary crop-livestock mixed (CLM) production system. This system is practised in the highlands (above 1,500 metres elevation) which cover about 40% of the country’s land mass, primarily in the regions of Tigray, Amhara, Benishangul Gumuz and major parts of Oromia and Southern Nations, Nationalities and Peoples (SNNP). The CLM system is based on limited communal and/or private grazing areas, crop residues, and naturally grown bushes and to some extent cultivated forage and industrial by-products.

2) Nomadic pastoral and agropastoral system. This system is practised in the lowlands (below 1500 m) in Somali and Afar regions and some parts of Oromia, SNNP and Gambella regions. The pastoral production system is based on extensive communal grazing.

3) Intensive specialized commercial livestock production. This system is practised in urban and peri-urban parts of the country. The intensive commercial system is based mostly on private grazing, cut and carry forage, industrial by products and formulated feed.

The two major small ruminant production systems(CLM and pastoral) and their sub-production esystms are mapped in Figure 1 based on administrative zone data. Mixed zones with both types of production system were assigned to the system that the majority of their districts belonged. The sub production systems include 1) CLM-predominantly cereal growing system where ruminant flock sizes are small and graze in communal grazing lands, as practiced in the northern and central highlands of the country, 2) CLM-predominantly *enset* growing system where ruminant flock sizes are small and tethered in backyards, as practiced in the southwestern highlands, 3) Pastoral-predominantly small ruminant livestock herds where ruminant flocks sizes are relatively large and are the dominant species as practised in the Afar and Somali regions in the eastern and south eastern lowlands of the country, and 4) Pastoral-predominantly cattle system where cattle are the dominant species in the pastoral herd and ruminant flocks’ size are relatively small, as practised in the southern lowlands of the country.

The intensive specialized small ruminant production system mainly consists of fatting units in urban and peri-urban areas and as such it is difficult to display on national scale maps. There is a lack of data about this system but generally this system can be considered relatively insignificant and is not considered further in this paper

**Supplementary figures**


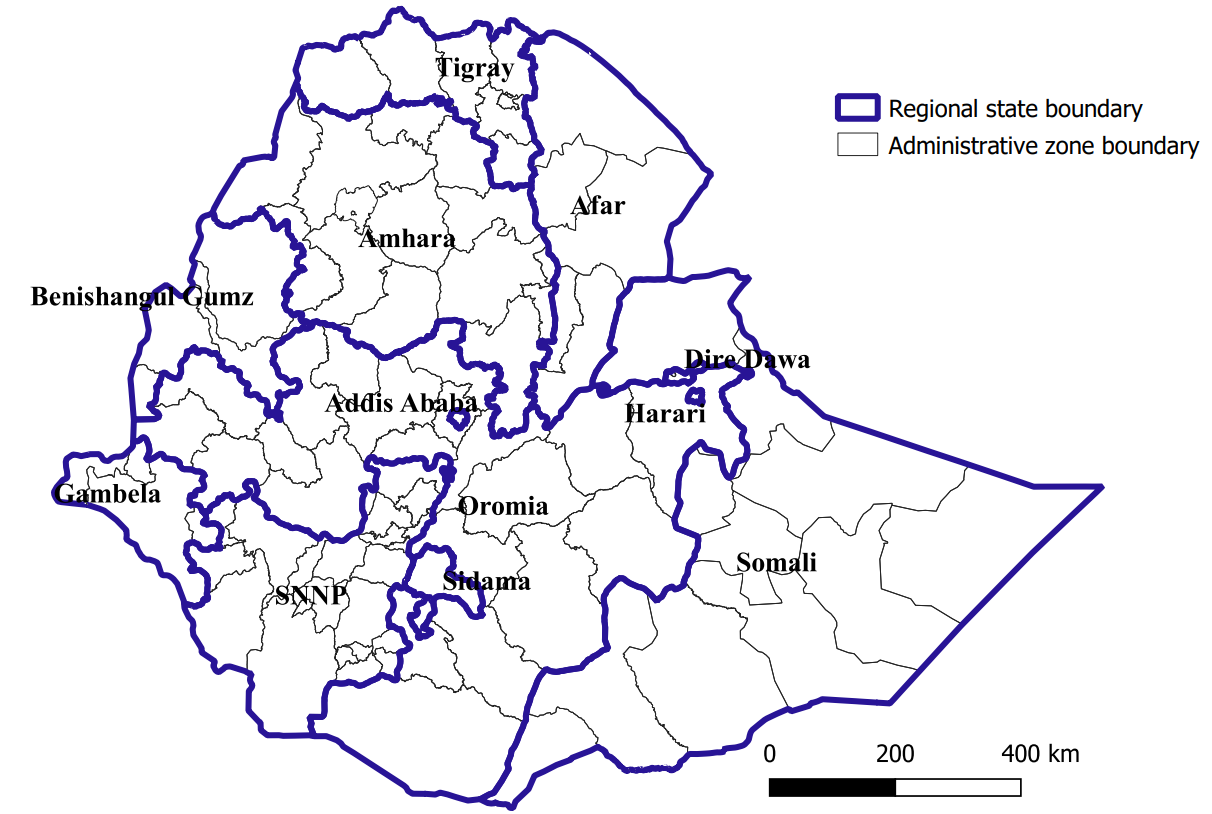


Figure S1. Administrative map of Ethiopia with region and zonal divisions


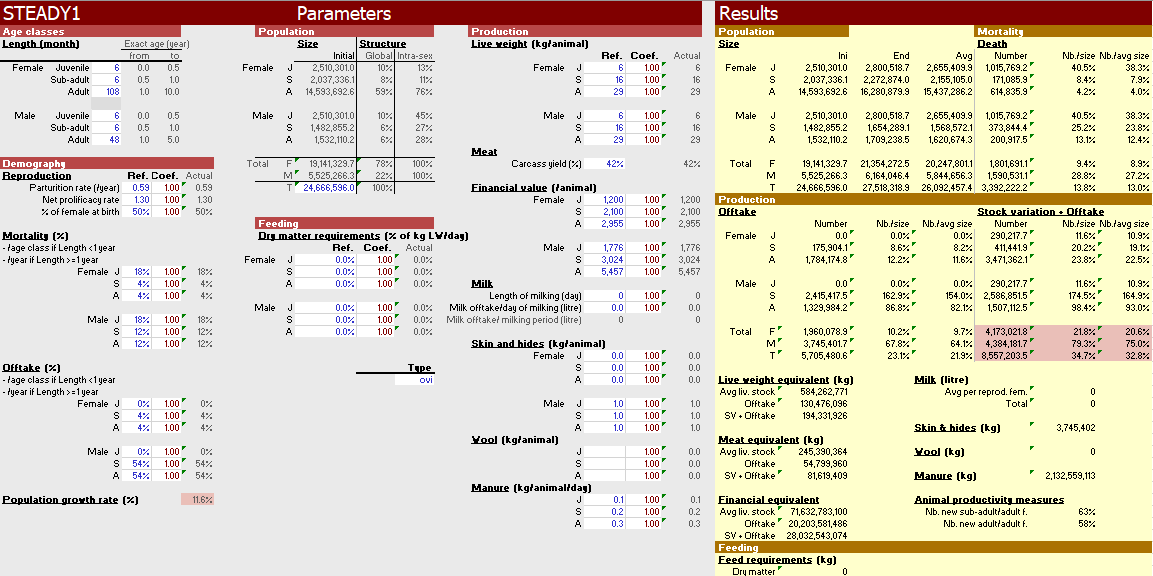


Figure S2. the DYNMOD herd model interface with data for sheep in the CLM system

**
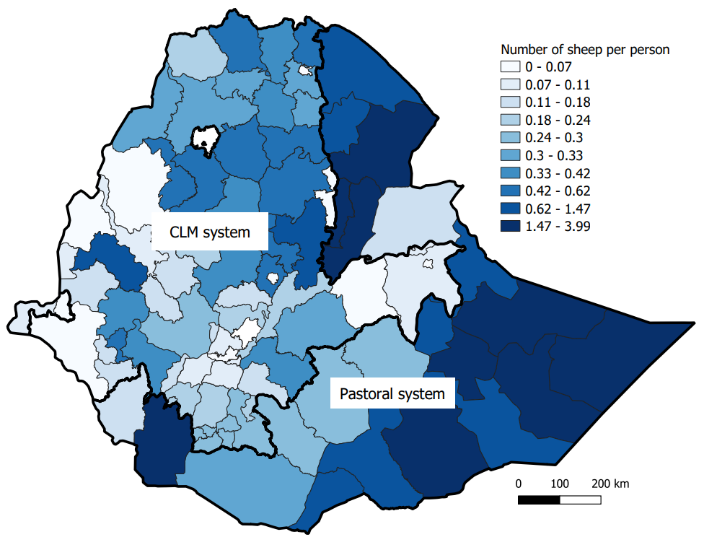

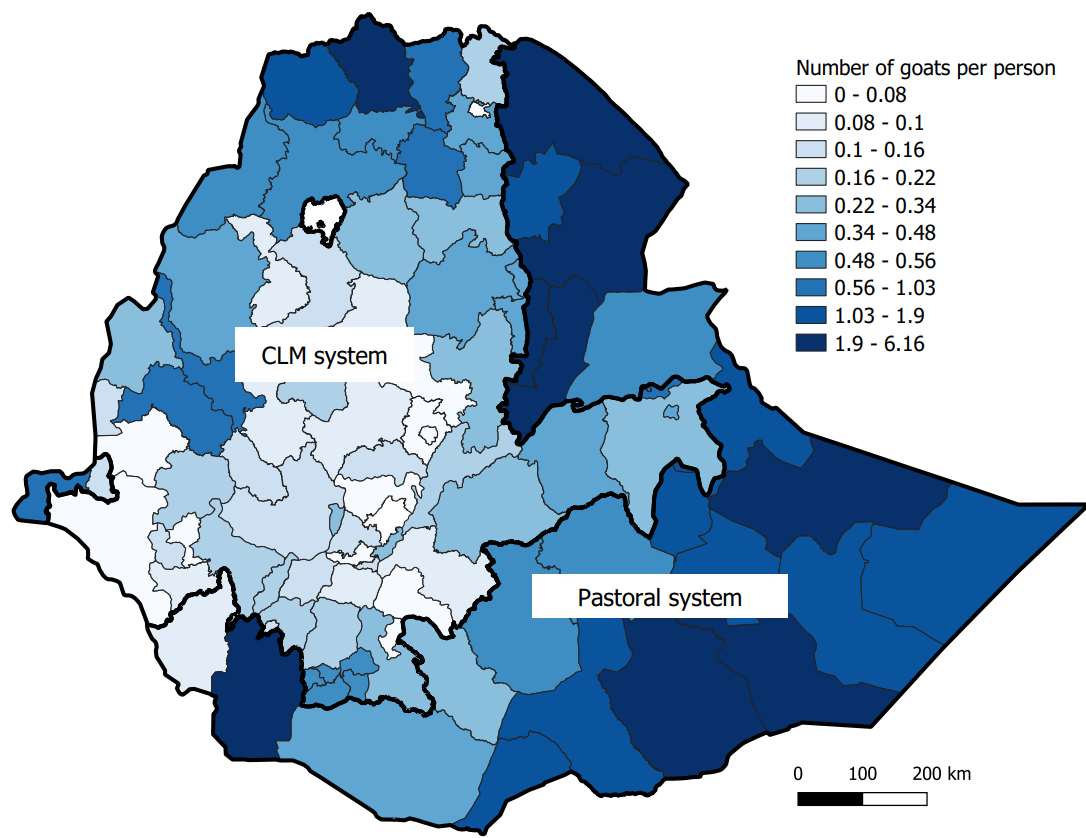
**

b

a

Figure S3. Distribution of small ruminants *a*) Number of sheep per person, *b*) Number of goats per person. The bold black line is border of CLM and pastoral systems

Figure S4. Flocks size distribution for a. sheep in the CLM system (N =12,090), b. sheep in the pastoral system (N= 6341), c. goats in CLM system (N=12643) d. goats in pastoral system (N= 7980). Note the difference in y axis scale between CLM and pastoral systems.

Figure S5. Flock structure of small ruminants by species and production system
